# “It Has Made Me Think”: Engaging the Public with the History of Health in the Modern Irish Prison

**DOI:** 10.1007/s10912-022-09761-2

**Published:** 2022-10-22

**Authors:** Catherine Cox, Oisín Wall

**Affiliations:** 1 School of History, University College Dublin, Dublin, Republic of Ireland; 2 Radical Humanities Laboratory, University College Cork, Cork, Republic of Ireland

**Keywords:** Prisoners, Public history, Health, Participatory arts-project, Exhibition

## Abstract

Since the establishment of the modern prison system in the early nineteenth century, prisons and prisoners have been construed as sites of moral, social, and biological contagion. Historic and contemporary studies show that most prisoners experience severe health inequalities, higher rates of addiction and mental health issues, and lower life expectancy than the rest of the population. They also come from deprived social strata. Yet, these aspects of Irish penal history have been largely neglected in academia and popular histories. Our article discusses two public history projects—an art installation, *The Trial*, and a museum exhibition, *Living Inside*—that engaged different publics with the long history of health and welfare in Irish prisons. Developed by the research team on the Wellcome Trust Investigator Award “Prisoners, Medical Care and Entitlement to Health in England and Ireland, 1850-2000,” based at University College Dublin, the projects adopted different methodologies to engage their audiences and explore the experience and management of health and welfare in nineteenth- and twentieth-century Irish prisons. We further examine the different methodological approaches of each project, their varied aims and audiences, and the impacts reported by audiences and participants. The article also considers some of the challenges of doing this kind of public history, both in terms of working with marginalized communities and presenting research about difficult subjects to various audiences.

In 2017, Michael Donnellan, Director General of the Irish Prison Service (IPS), described Irish prisons as the “asylums” of the twenty-first century (Pollak 2017). Such characterizations are not novel. From the establishment of the “modern” prison system across Ireland in the early nineteenth century to the ongoing COVID-19 pandemic, anxieties about prisoners’ physical and mental health have concerned state and prison officials, reform organizations, and prisoners (Porter 1995). Drawing on original research, this article discusses two public history projects—an art installation, *The Trial*, and a museum exhibition, *Living Inside*—that engaged different publics with this long history of ill health in Irish prisons, focusing specifically on physical and mental illness.

While prison populations experience a range of severe health inequalities, prison doctors, chaplains, and other staff as well as commentators from outside prisons have emphasized the high rates of psychiatric diagnoses and substance dependency (Cox and Marland 2018). In the nineteenth century, the problem was so acute that the Royal Commission on Irish Prisons criticized prison authorities for their failure to address the issue (1884, 32). A 2005 study found that 20 percent of male and 32 percent of female committals needed psychiatric assessment (Kennedy et al. 2005, “Summary”). Likewise, alcohol dependence has long been a feature of prison populations: in the 1970s, 42.5 percent of prisoners were categorized as excessive drinkers (Irish National Council on Alcoholism 1978, 12–3). Furthermore, in 1997, 66 percent of prisoners at Mountjoy Prison, Dublin, had used heroin (O’Mahony 1997, 103). Many prisoners have also suffered physical and sexual abuse and were confined as juveniles (Whitaker 1985, 31; Penal Servitude Acts Commission 1878–1879, 797).

The challenges these health inequalities present to the prison and to prison staff are significant. While the principle of equivalence specifies that prisoners are entitled to the same standard of healthcare as the general public without discrimination, many inmates enter prison in greater need of support and treatment (Niveau 2007). Yet, for much of the twentieth century, most Irish prisons had limited psychiatric services, while transfers to hospitals were slow (Prison Study Group 1973, 50–1, 57, 62, 67–8). These challenges are compounded further by inmates’ traditional distrust of doctors, who are often seen as an extension of the prison’s disciplinary apparatus (Cox and Marland 2018; Whitaker 1985, 65; Miller 2016).

Despite the complex relationship between illhealth, disease, and the prison environment, the topic has been largely neglected in penal histories of Ireland. Instead, histories of political prisoners and of female criminality have dominated (Beresford 1987; Farrell 2013; McConville 2002, 2014; Murphy 2014), despite both groups forming only a small proportion of the average daily prison population. In 2018, for example, female prisoners comprised only 4.2 percent of the Irish prison population (IPS 2018, 17–21). Today and historically, most prisoners in Ireland are men convicted of non-political activities.

Among historians in Ireland, there is a long-standing tradition of and enthusiasm for public history and for reaching audiences outside academia. In recent years, public history has been heavily inflected by the Decade of Commemoration (2012 to 2023), which marks several important centenaries from the Home Rule Bill (1912) to the end of the Irish Civil War (1923). These commemorations have made a sustained effort to explicitly incorporate the voices and experiences of actors, notably women and children, and events, such as the 1918 influenza epidemic, which had been largely excluded from previous commemorations. There have also been important public history interventions addressing the Northern Ireland conflict (known as *the Troubles*). Despite this vibrancy, scholarship on the nature and purpose of public history in Ireland is relatively nascent. Most studies replicate and reinforce the emphasis on the “national political narrative,” while activities that cover unrelated topics are largely side-lined (Cauvin and O’Neill 2017; Casserly and O’Neill 2017; Regan 2010).

A notable exception to these trends has been the rise of academic and popular interest in the institutionalization of marginalized and vulnerable populations in Irish workhouses, psychiatric institutions, industrial schools, and Magdalene Laundries, much of which concerns physical and mental welfare as well as institutional abuse. Today, national audiences are acutely aware of Ireland’s difficult and often abusive and violent history of institutional confinement, especially following Mary Raftery’s *States of Fear* documentary (1999) and subsequent official investigations into institutions of confinement (Pine 2011; Cox 2018). Research into these events has produced extremely important public history events, websites, and more, which act as “ethical remembering” of shocking instances of twentieth-century institutional violence that have been too often ignored by contemporary society (Pine 2011, 16). One example was Alison Lowry’s art installation “(A)Dressing Our Hidden Truths,” at the National Museum of Ireland (Collins Barracks, Dublin), which responded to Ireland’s non-penal carceral history. According to Lynn Scarff, Director of the National Museum, Lowry’s exhibition opened the Museum as a “platform for exhibiting work that challenges us and connects with some of the biggest issues of our day, including this traumatic past” (Whitty 2019, 5). Lowry’s exhibition and similar events attract sizeable audiences and are significant acts of unpicking instances of “social forgetting” in Irish history (Beiner 2018).

Yet, these histories of the institutionalized and the events that engage public audiences have elided prisons and prisoners. The reasons for this omission are many, varied, and complex. In contrast to other institutions, the prison remains in operation globally as the primary social, political, economic, and cultural response to criminal activities. While public opinion surveys in some jurisdictions indicate a diversity of attitudes as to whether prisons are successful in preventing crime, the continued growth of the global prison population suggests that prison complexes are unlikely to disappear soon (Walmsley 2016, 14; 2018, 17). For some, the prison is a necessary institution to contain moral, social, or biological contagion. In this context, discussing the physical and mental health of prisoners with public audiences poses specific challenges. Citizens are often unfamiliar with the health inequalities experienced by people prior to their imprisonment and are unaware of the welfare and medical services provided in prisons. A vocal minority advocate that such services be kept to the minimum (Conlon 2017). As perpetrators of crime, prisoners are often constructed as a transgressive and deviant population and, consequently, do not fit into an accepted narrative of vulnerable subjects of institutional neglect and ill treatment. Also, in contrast to other jurisdictions, Ireland’s prison population, especially in the twentieth century, has been relatively small, falling below 400 people in the 1950s, and consequently could be easily and conveniently forgotten. Finally, public audiences often disengage from exhibitions about stigmatized issues (Dudley 2017, 194), and the stigma around prison is so significant that a former prisoner might “never be viewed as a normal citizen again” (Hilliard 2019).

Undeterred or perhaps spurred by such challenges, between 2016 and 2019, the team on a Wellcome Trust-funded project entitled “Prisoners, Medical Care and Entitlement to Health in England and Ireland, 1850-2000” developed several public engagement events on the history of health and prison medicine in the nineteenth and twentieth centuries, with a focus on the individual experiences of those incarcerated. This article discusses two projects developed at the School of History, University College Dublin (UCD): *The Trial* (2018), a multi-screen audio-visual art installation, and an exhibition called *Living Inside: Six Voices from the History of Prison Reform* (2019). Both projects linked specific historical findings on prison medicine and the health of nineteenth- and twentieth-century prisoners with contemporary experiences of psychiatric, psychological, and physical distress in Irish prisons, but they adopted very different methodologies. Drawing on our perspectives as academic historians, the article examines and evaluates these disparate approaches and discusses the challenges and opportunities of engaging with the public and with experts by experience. We also reflect on our ethical responsibilities when working with vulnerable groups and to our audiences when dealing with difficult histories.

*The Trial* and *Living Inside* were installed in the Kilmainham Gaol Museum, Dublin, and the choice of venue influenced the form and development of each piece. Opened in 1796, Kilmainham is famous as the prison where many of those valorized by the Irish nationalist tradition were incarcerated or executed, although most inmates held there had not committed politically motivated crimes (O’Sullivan 1996). In 1924, Kilmainham was decommissioned as a prison and fell into dereliction; however, due to its pedigree as a nationalist shrine, it was opened as a museum in 1971 (Cooke 2000; Zuelow 2004; McAtackney 2016). The museum includes Kilmainham County Courthouse, a local court that operated from 1825 until 2008 and was opened to the public in 2018. Today, Kilmainham Gaol Museum is the largest prison museum in Europe with a diverse international audience of over 400,000 visitors each year.

## *The Trial*

*The Trial* was a multi-screen video and sound art installation that engaged the senses to explore mental health and human rights in the Irish criminal justice system, past and present*.* The work was installed in April 2018 for two weeks in the Kilmainham Courthouse and was viewed by an estimated 21,000 people, including experts from the criminal justice system, policymakers, cultural workers, community activists, and ex-offenders. A site-specific artwork, it drew inspiration from the trials held in the courthouse. *The Trial* received very positive feedback and reviews, and, between July and December 2019, it toured to Spike Island, Cork; Lifford Courthouse, Donegal; and Dublin Castle, where it was programmed as part of the Smashing Times Arts and Human Rights Festival Dublin (McCann 2019).

The principal historian on *The Trial* was Catherine Cox, whose research focuses on the mental health of adult prisoners in nineteenth-century Ireland and England and who is the Principal Investigator on the Wellcome Trust project. The lead artist was Sinead McCann, a visual artist with extensive experience of working with vulnerable and marginalized groups using creative participatory methodologies to develop collaborative artworks exploring and representing social issues. The production also drew on research by Fiachra Byrne, a Postdoctoral Fellow on the Wellcome Trust project, whose work examines the mental health of juvenile prisoners. While the academic research that featured and fed into the production of *The Trial* was funded through the Wellcome Trust project, in 2017, McCann secured additional funding from the Arts Council of Ireland to develop the artwork.

When planning the piece, Cox and McCann had a series of overlapping aims and sought to develop a process with a democratic epistemology. They aimed 1) to produce and communicate history; 2) to develop collaborations with people who have experience of prison medical services and to introduce them to historical research materials in creative participatory workshops; 3) to collaborate with other socially engaged creative practitioners. Finally, Cox and McCann aimed to make a challenging piece of art that would reach significant audiences. To achieve these goals, McCann developed a creative participatory project that directly involved historians, other creative partners, and ex-offenders availing of the services of the Bridge Project, Dublin. The Bridge Project is a community-based alternative to custody for adult males with a history of repeat violent offending, and, with a full probation team, it supports people on release from prison. Cox and McCann also worked with prison staff, medical and lay representatives from the IPS, and the Irish Penal Reform Trust (IPRT). Through collaborations, the production process adopted a bottom-up methodology, which facilitated co-production and co-authorship, and it was through this collaborative process that the *story* being told in the artwork was formed. The methodology valued the input and expertise of all partners and emphasized processes and relationships. Consequently, the collaboration with and impact on partners and participants was as important as the final output. While the narration of specific historical events or phenomena was important, it was not the primary goal. Early in the process, McCann decided on the artwork’s form; however, the specific content emerged during the production process.

**Fig. 1:**
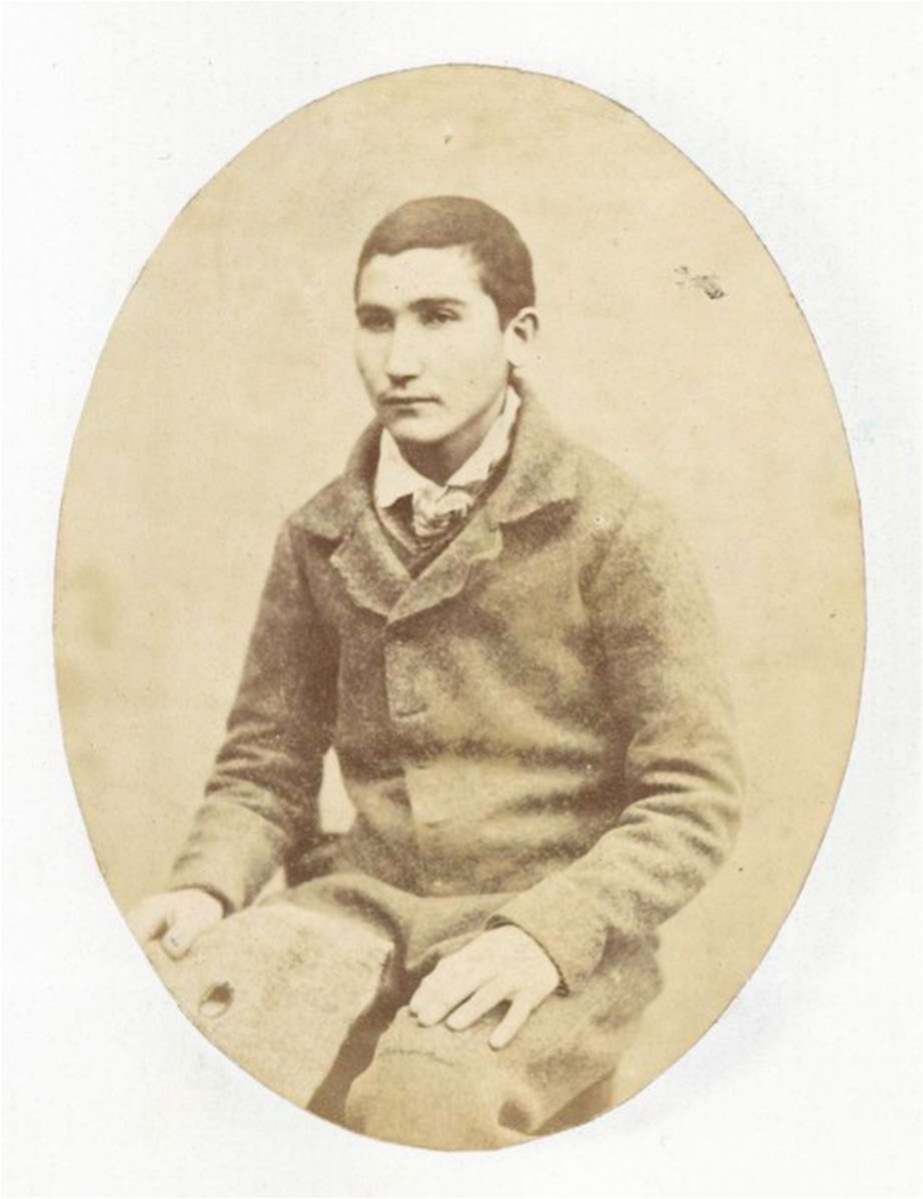
“Photograph #3 [Patrick Downs?].” Thomas A. Larcom Photograph Collection, Volume 1, August 1857, MssCol 1694. Image from the New York Public Library Digital Collections. http://digitalcollections.nypl.org/items/510d47dc-95cb-a3d9-e040-e00a18064a99

The project’s success depended on convincing a group who used the Bridge Project services to participate and share their experiences of illness and healthcare in prisons. Working with such a group posed risks to the project; for most, availing of the Bridge Project services was voluntary, and there was the possibility that participants might disengage from the project, especially as it placed a heavy demand on their time, for which they were not financially remunerated. To recruit participants, McCann liaised extensively with the Bridge Project probation team to identify people with relevant experiences and who were willing to discuss their personal histories. To convince them that it was worthwhile, Cox and McCann pitched the idea of the project to potential participants in an introduction session, showing the group a range of visual and textual material from Cox’s and Byrne’s original research. When selecting these materials, it was necessary to consider participants’ literacy and language skills, especially when using Victorian examples. For this session, the material was primarily visual and included photographs of accommodation at Spike Island Prison, Cork; St. Patrick’s Institution for Juveniles, Dublin; and Mountjoy’s infamous basement cells in 1985. Other selected material included images of “suicide nets” or “safety wires” used in prisons to prevent suicides (General Prisons Board 1886) and a photograph of Patrick Downs, whose image is included in a volume of rare photographs of prisoners held in Mountjoy Prison in the 1850s (see Fig. 1).

While some of this material does not directly address the issue of health in prison, its use during the introductory workshop helped to identify topics that resonated with potential participants. By facilitating a bottom-up process during the first workshop, some of the attendees almost immediately shared their experiences of health and sickness in prison. One attendee recalled his attempt to hang himself while in solitary confinement in Mountjoy. Other members were quiet, and some were downright skeptical. We, Cox and McCann, had not anticipated the level of openness displayed by some, nor were we prepared for it in terms of recording these contributions. We had been concerned the topic would prove too intimate and our questions too invasive, and we did not know in advance whether the men had undergone periods of psychiatric or physical illness while incarcerated. In the end, six men who had spent decades in prisons across Ireland agreed to participate and their accounts covered the period from the 1970s to the 2000s. At the introductory session, certain themes emerged from the discussion that resonated with the topics covered in our historical research. This included high rates of mental distress and instances of self-harm while incarcerated; diverse responses from prison medical staff and, in some instances, difficulty in accessing appropriate medical treatments; problems associated with prison diet; and a history of incarceration as juveniles prior to periods of imprisonment.

Following this session, Cox and McCann commenced a three-month series of weekly three-hour creative workshops with the participants. Cox participated in the first set of workshops, which centered on historical research materials that responded to issues the men raised during the early sessions, while McCann integrated historical materials into later workshops. For these creative workshops, Cox selected additional historical case studies. These included correspondence between the General Prisons Board and staff at Galway Prison about prisoner John Burke, who, protesting the diet in Galway Prison, attempted suicide, prompting an inquiry into practices at the prison in 1885 (General Prisons Board 1885). To contextualize this case, the men were shown the 1882 General Prisons Board official prison dietary scales and rules, which detailed the allocation of diet to different classes of prisoners and convicts. As many of the men had been in St. Patrick’s Juvenile Prison, Cox and McCann also showed them a 1963 Radharc video documentary on the institution as well as extracts from a 1963 confidential report requested by Charles Haughey following allegations of beatings and mistreatment made by four former inmates of St. Patrick’s. These materials prompted detailed discussions among the group, and members opened up about their experiences of psychiatric and physical illnesses in prison and the medical treatment they had received. They narrated experiences of being in solitary confinement for prolonged periods; self-harm and suicide attempts; the obligation they felt to “watch” other inmates for signs of depression and distress. They also discussed transfers to the Central Mental Hospital (CMH), Dublin, and they revealed the psychological impact of being separated from family and memories of incarceration in industrial schools and in St. Patrick’s Juvenile Prison.

**Fig. 2:**
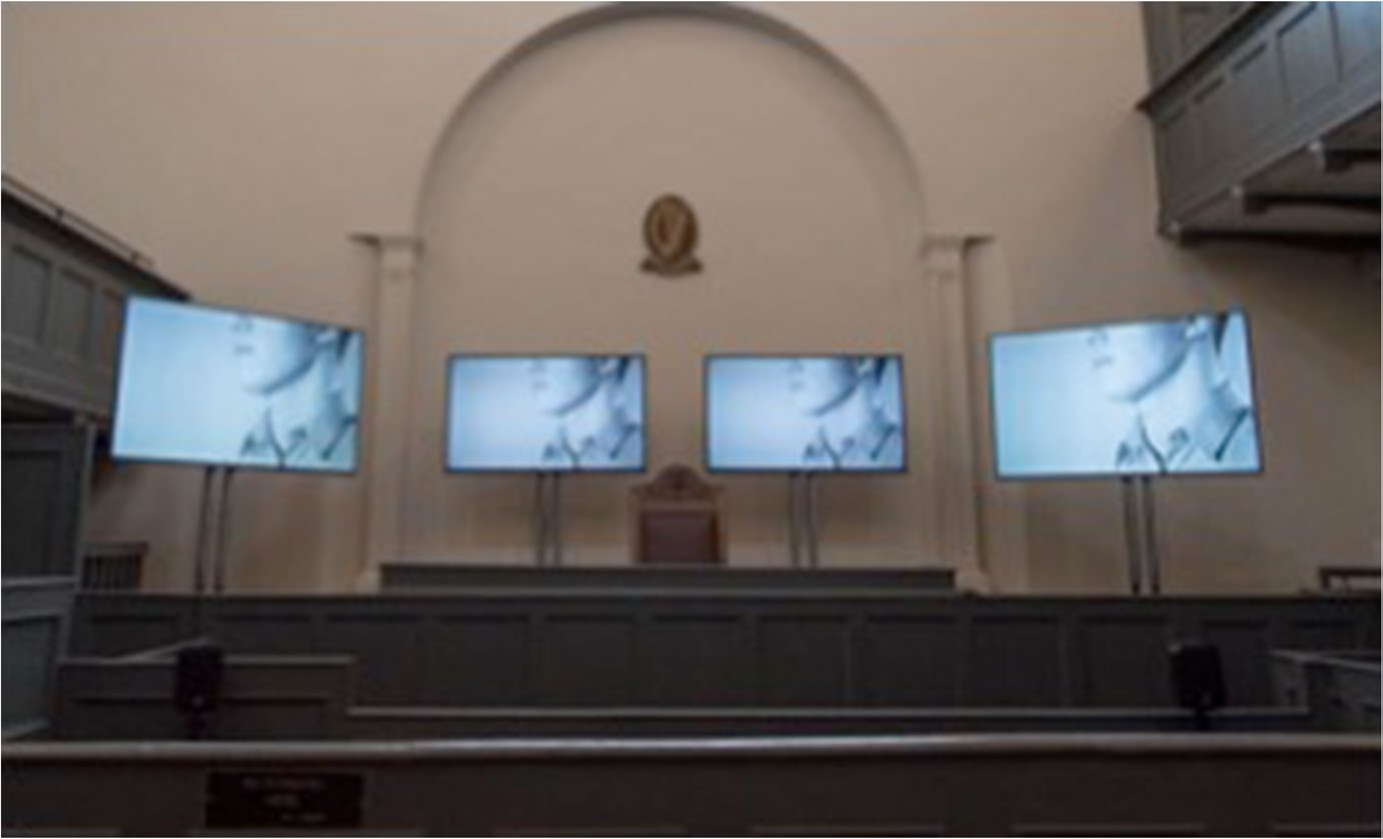
Four-channel synced video and sound installation in *The Trial* at the Old Courthouse Kilmainham Gaol Museum, Dublin. April 2016. Artwork by Sinead McCann. Photography by Conor Mulhern

Drawing on these discussions, performance-based workshops, and one-on-one interviews with McCann and supported by specialist scriptwriter Sarah Meaney, participants authored mini scripts or monologues about their experiences and responses to the historical research. McCann shared these monologues with medical and lay people working in the criminal justice system including two prison governors, a chaplain, an addiction counselor, and IPS and IPRT representatives to capture their reflections and input. The final script incorporated their responses, alongside the men’s writings and extracts from the historical case studies. McCann then developed the audio and video installation over the next three months, regularly meeting with the participants and the historians, who commented on drafts of the script and video production. An important part of the project was to include an actor who had spent time in prison. Tommy O’Neill, who had been in prison in Ireland, agreed to participate in some of the creative workshops with McCann and the men. His experiences were featured in the script, and he performed in the final installation (see Fig. 2).

Working on a participatory project involving marginalized groups entailed specific challenges. Cox and McCann were very conscious of their ethical responsibility to the ex-offenders as a vulnerable group and aware that this type of engagement can have both negative and positive impacts (Whelan 2014). Weekly debriefs were held with the Bridge Project staff, which alerted probation staff to the men’s emotional responses during or after workshops and ensured they had access to counseling services. Cox and McCann also carefully managed the men’s expectations for their involvement in the process, making it clear that they would not advocate for specific legal campaigns for compensation or redress, and any benefit they might experience would be gained through the process of participating. Pre-, mid-, and post-project questionnaires, one-on-one interviews, and group conversations were held throughout the process to capture and manage the men’s expectations, challenges, and the impact of our work. In this respect, the project differed from academic involvement in campaigns to secure redress for victims of institutional abuse, such as the “Justice for Magdalenes” research project at UCD.

Cox and McCann also had to prepare participants for the moment when the artwork was displayed in the public arena. During the development process, participants had engaged in relatively private discussions of sensitive information among trusted partners, viewing earlier iterations of the artwork on laptops at the Bridge Project premises. Once installed at Kilmainham Courthouse, however, their stories would be heard and viewed in public on four 70-inch screens. Aware that this would be a very different experience for participants and that it might elicit strong emotional responses from them, the team decided to give the men time to absorb the artwork in private. This process was managed in stages. Prior to the public opening of *The Trial,* the men and their families attended a private screening. This was followed by a private launch screening for other project partners, representatives from the IPS and NGOs who contributed to the script, and guests from a range of sectors and academic disciplines. Following that screening, the audience attended a panel discussion, which featured Cox, McCann, representatives from the Bridge Project and the IPRT, and one of the participants. The other participants and their families contributed to the discussions as audience members.

Additional challenges also emerged in terms of the specific subject covered in the artwork. Through careful facilitation, those with experience of being in prison shaped and directed the topics covered during the production process, rather than Cox or the creative arts partners. As a result, it was not possible to control content, as was done during the development of the exhibition *Living Inside* (as will be discussed below). Instead, Cox and McCann could only nudge the project towards themes they wished to address. For example, mental health was not intended to be the main subject. However, its prominence in participants’ stories made it a dominant thread throughout the piece. Furthermore, although Cox and McCann hoped to access the experiences of a diverse range of prisoners, they were slightly disappointed not to include women’s stories; however, given the relatively small number of women in Irish prisons and the considerable attention this group has received, they knew their experiences were told elsewhere. More problematic was the inability to draw, in greater depth, on the Traveller community’s differentiated experiences of incarceration. Studies show that Travellers are committed to Irish prisons in disproportionate numbers, experience discrimination when confined, and have high rates of psychiatric diagnosis (Irish Penal Reform Trust 2014; Doyle 2017, 20). While a Traveller man participated in the project, and his story features in the installation, for complex reasons, he did not engage in the production process as frequently as the other men. Despite McCann’s careful consideration of sampling and representation at the early stages of the project and repeated efforts to involve women and members of the Traveller community, these experiences are underrepresented in the piece.

There are significant benefits for historians in pursuing creative participatory projects to engage audiences in public debates about the health rights of groups such as prisoners, who may struggle to attract attention and understanding from audiences. The processes and practices pursued in these collaborations move beyond a one-way hierarchical process of dispensing information and allow for exchanges of knowledge among all involved. The material that formed the basis of the script was researched and produced jointly by the participating men with experience of healthcare in prison, historians, artists, and others. Consequently, *The Trial* included verbatim extracts from the men alongside the historical research material and was a form of “verbatim theatre,” which, as Robin Soans (quoted in Wake 2013, 332) argues, provides words “with listening ears” and amplifies them. The combination of historical materials and contemporary experience strengthens the piece significantly. As one anonymous viewer reported, “I worked in the prison system for 40 years, & it was a powerful portrayal” (Anonymous, comments from artwork feedback card, 2018, Dublin). Also, by bringing the past and present together, audiences were informed of the long history of the relationship between prison, disciplinary regimes, and mental health and learned about current practices and conditions in Irish prisons. As such, they were prevented from falling back on assumptions about historical progress and dismissing past issues as no longer relevant and now resolved.

The post-production evaluation conversations with participants revealed the positive results of using history in these projects. The participants reported that they enjoyed learning about prison history and found it helpful for opening up discussions of private and personal issues. They described how history gave their experiences “more weight” and helped them feel heard and believed. As one of the participants reported: “This is the first time I have ever been heard, that I have ever spoken about my experience; I got to tell my story, from the age of 12 upwards from the time of reform school, borstal and time in prison” (Anonymous, comments from post-production evaluation conversations, 2018, Dublin). They also reported that it allowed them to respond to historical documents with the benefit of personal experiences. As Karen Harvey (2015, 539) observes, the past “provides a satisfying space for identity formation, creative thinking and imaginative reconstruction.” By situating their narratives within historical contexts, the men reported that history helped them narrate their experiences, revisit memories and topics difficult to recall, and reconnect with family and society. They also reported that the involvement of professional academic historians in the project gave cultural capital to their experiences and stories. As one man mentioned: “I felt like ‘somebody’ when I was at the launch, and seen [sic] the artwork that I had a part in making, and everyone there to watch. Instead of feeling like ‘no-body.’ I felt part of something important, like a part of other people’s lives, a part of the bigger society” (Anonymous, comments from post-production evaluation conversations, 2018, Dublin).

## *Living Inside*

**Fig. 3:**
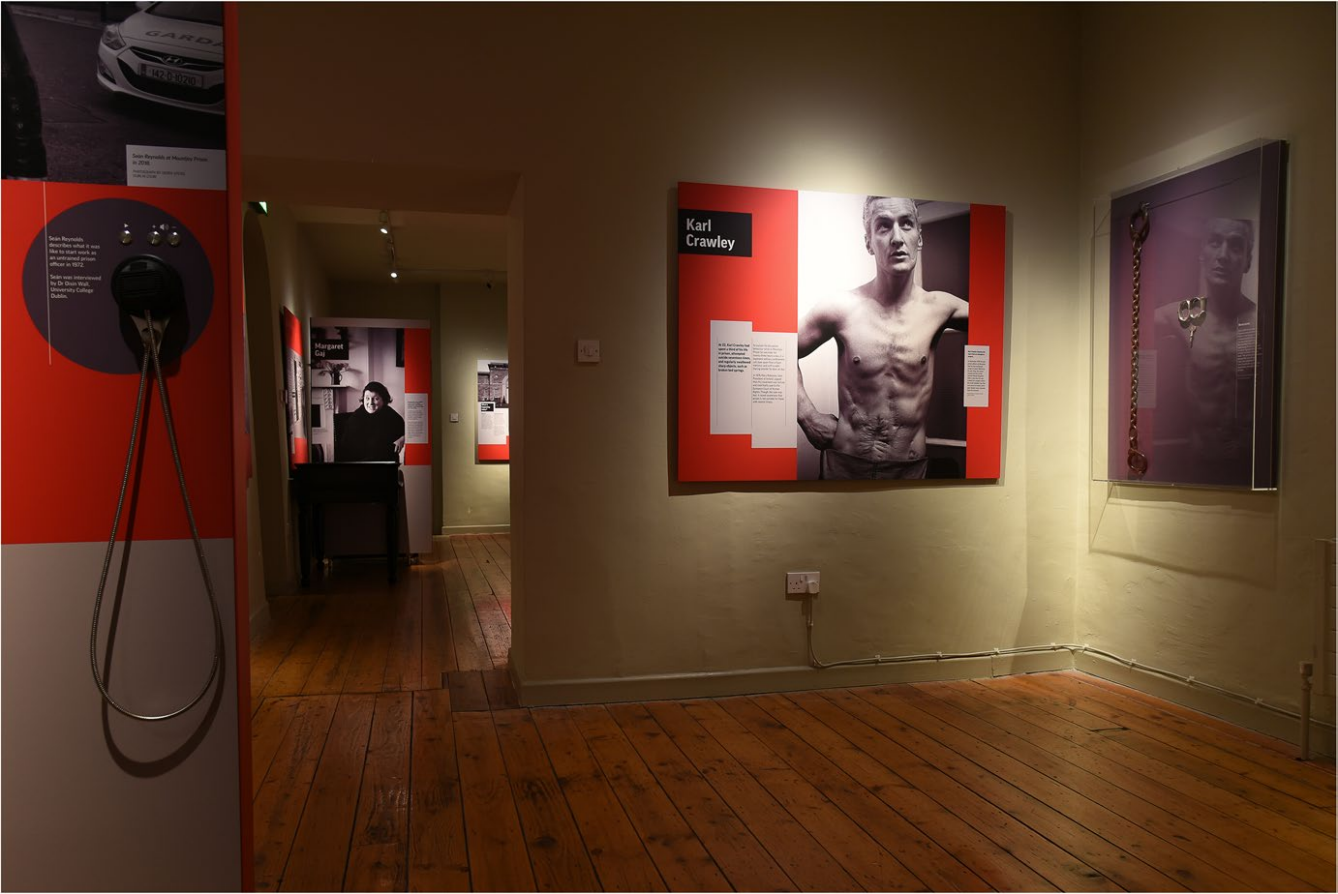
The exhibition combined oral histories, photographs, and historical objects to tell six stories. *Living Inside* at the Kilmainham Museum, Dublin. 2019. Photography by Conor Mulhern

**Fig. 4:**
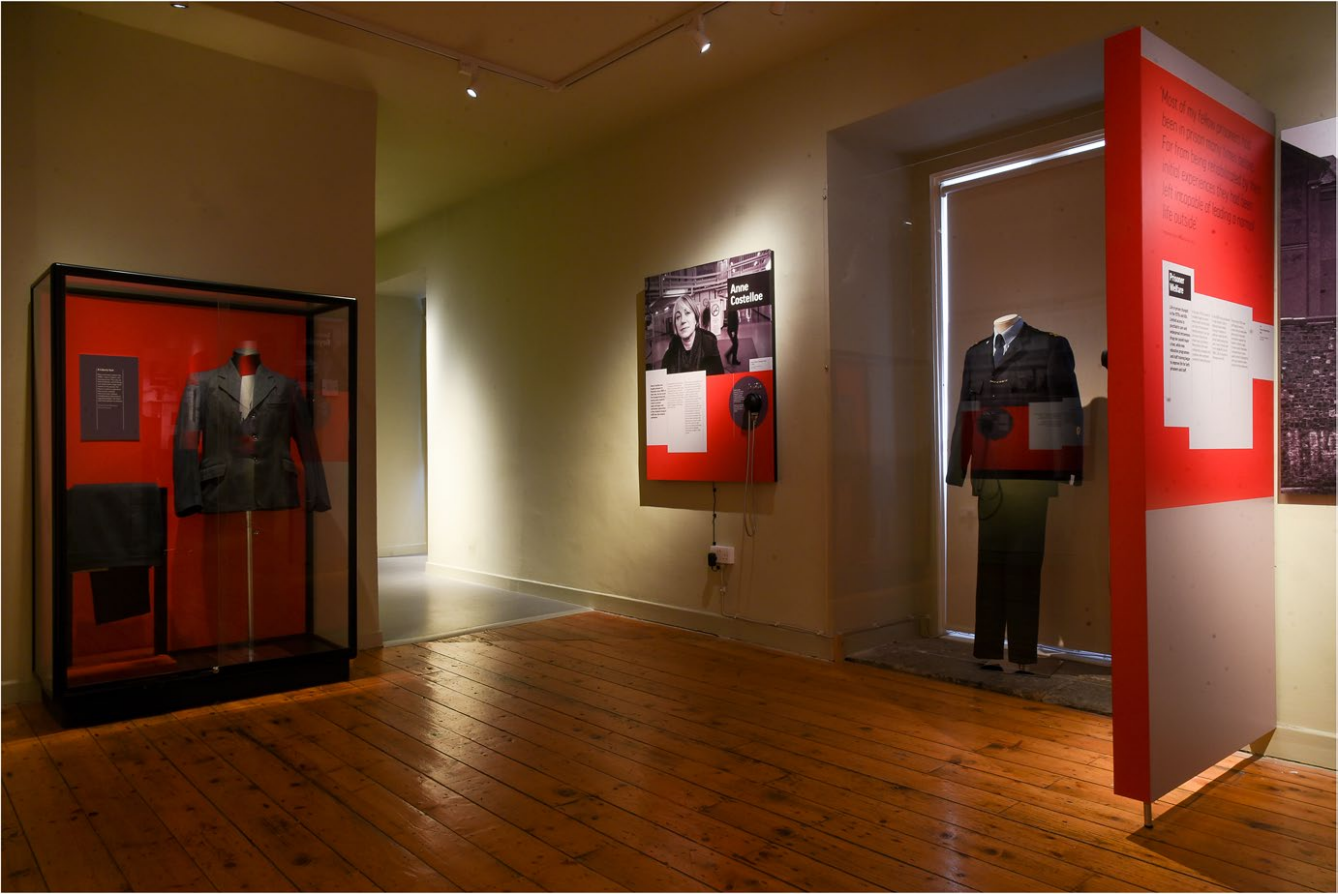
Portrait photography and personal objects, like clothes, emphasized the focus on individuals’ stories. *Living Inside* at the Kilmainham Museum, Dublin. 2019. Photography by Conor Mulhern

*Living Inside: Six Voices from the History of Prison Reform in Ireland*, the second project covered in this article, was an exhibition that ran from February to July 2019 at the Kilmainham Gaol Museum. It focused on the experiences of six people involved with prison reform and welfare since the late 1960s, which included three prisoners, two staff members, and a prisoners’ rights activist. Oisín Wall was the lead historian on the exhibition, and his background in museum curation added specific expertise to the project. Catherine Cox and Sinead McCann—who, separate from her role as the artist on *The Trial*, was a public engagement officer on the Wellcome Trust project—provided support, working with Wall, Ann Scroope of Scroope Design, and Wendy Williams of Wendy Williams Design. The exhibition also featured the work of photojournalist Derek Speirs, whose images, as seen in Figs. 3 and 4, draw audiences’ attention to the subjects’ humanity. The exhibition was based on Wall’s research into prison reform movements in late twentieth-century Ireland. The primary aim was to effectively communicate research on penal reform from the 1960s and demonstrate how prisoners’ mental and physical health and the low standard of prison medicine were integral to these campaigns. There were, however, challenges in representing, for a public audience, the often-troubling recent history of health and welfare in the Irish prison system.

Audiences often view content in light of their own diverse and sometimes traumatic experiences; when presenting an exhibition to a diverse audience, it was important to be aware of the potential impact of the work presented on audience members’ mental health. *Living Inside* included discussions of several potentially distressing subjects, such as suicide, self-harm, drug addiction, HIV and AIDS, death, and non-consensual psychiatric treatment. Each of these subjects on their own might prove difficult to discuss, but, taken together, they risk alienating or provoking distress in audiences. A second challenge is that these subjects had the potential to elicit a hostile reaction from audiences, inhibiting them from engaging with the exhibition at all. When beginning the curation, Cox and Wall were concerned that some visitors would adopt an anti-prisoner position that was so extreme it would render reflection impossible. Only a few visitors responded this way, with a very small minority making hostile notes on the exhibition feedback cards such as “prisons are too soft” and “don’t do the crime if you can’t do the time” (Anonymous, comments from exhibition feedback card, 2019, Dublin). However, these were not representative of most of responses. A final challenge of presenting these subjects to a diverse audience was communicating the everyday realities of life in prison, which can be an insular community. For instance, audiences unfamiliar with prison environments may commonly understand self-harm as an indicator of mental distress; as such, they may struggle to understand that, inside prison, such practices can be attempts to disrupt the order of the prison and are disciplinary as well as medical issues. Wall’s research highlighted accounts by prisoners who described their self-harming behavior as a conscious “project against authority,” expressing agency when they were otherwise powerless (Smith 1976, 18).

From the outset, we—Wall, Cox, and McCann—decided to turn these challenges into guidelines for how to curate the exhibition and engage visitors in “ethical remembering”: that is, to remember and perform remembrance “in order to create a more just future” (Pine 2011, 16). The ethical guidelines for this act of remembering were to design the exhibition in a way that minimized visitors’ alienation from the subject and sought to address themes that would engage visitors from a wide variety of demographic and geographic groups.

One of the focal points of Wall’s research is the Prisoners’ Rights Organization (PRO), a group that campaigned for improved prison conditions in the 1970s and 1980s. In 1980, the PRO helped convince the United Nations Congress on the Prevention of Crime and the Treatment of Offenders to include prisoners’ representatives, and in 1984, the group pressured the Irish government to convene the first public inquiry into the prison system. They also shifted the public discourse away from the relatively common representation of prisoners as subhuman or unworthy of concern. The PRO encouraged the view that prisoners were normal people, shaped by complex situations, who deserved a chance at rehabilitation or, at least, fundamental human rights. In 1973, a well-publicized letter from Prisoners’ Union leaders, which became foundational to the PRO’s campaign, stressed that “The most frustrating aspect of our few demands is that they are for basic human rights, nothing more” (Anonymous 1973a). The PRO achieved this shift in attitude by highlighting the relatable and troubling experiences of individual prisoners, which often revolved around health and healthcare. For example, their 1974–1975 campaign highlighted a cluster of suicides in custody to argue for improved psychiatric assessment and care in prisons. They smuggled out prisoners’ letters highlighting poor prison conditions, held press conferences with prisoners’ families, supported court cases against the state taken by prisoners, and took part in and publicized the reports of coroners’ inquests when prisoners died. The effect was remarkable, and by the late 1970s, the discourse about ordinary prisoners had been turned on its head (Wall 2019).

A modified version of the approach adopted by the PRO was used in the exhibition. It was decided that communicating a very focused idea in an emotionally engaging way would be the most effective way to communicate with skeptical visitors. After discussing and rejecting several possible messages, we decided that the exhibition’s core message would be that prisoners are human. This may seem simplistic; however, given the visceral reaction that audiences can have towards contemporary prisoners and the challenges many prisoners have had accessing basic human rights, it was important to lay a strong shared foundation. Building on the PRO’s tactics, individuals’ stories were deployed as a vehicle to communicate the history of prisons in this period while simultaneously highlighting the relatable, human experience of prisoners. After much debate, the content was winnowed down to the stories of six people whose experiences spoke to broader trends in the history of prison reform. These stories focused on visceral experiences of mental and physical illness, loss, and injustice, to which visitors, who may have had similar experiences outside prison, may be able to relate. This was effective: one visitor remarked that they found a story particularly engaging because, like the prisoner in it, they, too, had experienced mental health difficulties. Another visitor engaged with a story about a prisoner dying of AIDS because their friend had recently been diagnosed with HIV.

Each of the six stories in the exhibition recalled nationally important historical events through the stories of people who might otherwise have been elided from history. These stories were told, where possible, in each subject’s own words or those of their friends. This was achieved by including in the exhibition direct quotations, a reprint of a poem written by a man in the AIDS separation unit of Mountjoy, and three oral histories collected by Wall and Janet Weston, another postdoctoral researcher on the Wellcome Trust project. Throughout the exhibition, contextual and portrait photographs by Speirs were used to illustrate the stories. The exhibition also featured objects borrowed from Mountjoy Prison Museum and the Royal College of Physicians of Ireland that spoke to everyday prison life. Each story explored how the prison system has changed, the improvements and mistakes that have been made, and the very real effect that those changes have had on people’s lives.

To ensure the experiences of prisoners would be prominent, three of the six stories were about them. The first explored attempts by prisoners to organize and demand reform, highlighting both the poor prison conditions in Ireland in the 1970s and prisoners’ limited agency within the system. It focused on Danny Redmond, a founding member of the Portlaoise Prisoners Union in 1973 who led a nine-month campaign of labor strikes and sit-ins demanding reform of the prison system (Anonymous 1973c). This campaign tried to “highlight the lack of proper educational, medical and recreational facilities in the prisons” and demanded structural reforms (Anonymous 1973b; Anonymous 1974). Ultimately, however, the Union was broken, and Redmond was transferred to the Curragh Camp, a military prison where he served the remaining years of his sentence (Anonymous 1973d).

The second story was that of Karl Crawley and addressed prisoners’ individual struggles with the prison system and the lack of psychiatric healthcare in the 1970s. As a young man, Crawley suffered from severe mental health issues, and, by 1976, he had attempted suicide 17 times and had been transferred to the CMH 12 times (Anonymous 1975a). He received little psychiatric treatment in prison, and the prison medical officer admitted that sedatives were used intentionally to keep Crawley “at a low level” and to stop him from being disruptive (Davis 1976, 21). Later, Crawley told a journalist that he had once been sedated for so long that he had to relearn how to walk (Kerrigan 1996, 314). By the mid-1970s, Crawley was addicted to drugs, due in part to these long periods of sedation, an addiction that ultimately led to his early death at the age of 47 (Kerrigan 2003, 138). While confined, Crawley often climbed onto the roofs of Mountjoy Prison or the CMH to protest his treatment (Anonymous n.d.). Due to his psychiatric condition and disruptive behavior, he was held in the solitary punishment block in the basement of Mountjoy Prison and handcuffed whenever he left his cell. In the mid-1970s, he took cases to the High Court in Ireland and the European Commission on Human Rights, alleging that his treatment in prison amounted to torture. His petition was denied for procedural reasons, but the reporting of his case had an important impact on how the Irish public thought of prisoners’ mental health.

The third story dealt with the death of Derek Cummins, and it explored the experience of epidemic disease in prison and the impact that it could have on those outside as well as inside the prison. In 1986, Cummins died of AIDS-related pneumonia hours after being moved from Mountjoy Prison to the Mater Hospital (Anonymous 1986). Derek’s brother and sister, who had also been in Mountjoy, died of AIDS shortly after (Mara De Lacy 2016, unpublished interview by Janet Weston, November 17, 2016, Dublin). As Weston and Berridge (2018, 16) show, Cummins’ experience was, in many ways, typical of many families during the simultaneous heroin and AIDS epidemics that swept through Dublin in the 1980s. At the time, 40 people (about ten percent of prisoners in Mountjoy) had been diagnosed with HIV, primarily contracted through intravenous drug use (Anonymous 1986). Cummins’ death in 1986 and the accompanying public reaction forced the Irish government to acknowledge the depth of the HIV problem in Ireland and to launch its first awareness campaign (Power 1986).

Having told these stories, the exhibition moved on to show that others cared about and argued for prison reform. The fourth story dealt with how activists outside prisons adopted the prisoners’ cause and how prisoners’ rights became intertwined with other social movements emerging in Ireland. This was the story of Margaret Gaj, a well-known activist and restaurateur. Gaj was a founder of the Irish Women’s Liberation Movement, the Dublin Housing Action Committee, and other important civil rights groups in Ireland in the 1960s and 1970s. She was also a founding member of the PRO and one of its leading activists.The exhibition explained how Gaj and five other activists were arrested outside the trial of Karl Crawley, where they were demanding better psychiatric care for prisoners. Gaj was sentenced to a year in prison under a law which was intended to deal with subversive groups, like the IRA (Irish Republican Army). With the help of Mary Robinson, a barrister and later President of Ireland, the activists won their appeal against the sentence and the ensuing public debate brought prisoners’ rights firmly into the Irish popular discourse (Anonymous 1975b).

Focusing solely on prisoners and activists risked demonizing prison staff. To prevent this, the final two stories focused on prison staff members. One was about Seán Reynolds, a prison officer in Mountjoy from the 1970s to the 2000s. Reynolds’ oral history included rich details about how things had changed for prison officers over the decades, through both policy shifts and union activism (Seán Reynolds, unpublished interview by Oisín Wall, September 19, 2018, Mountjoy Prison, Dublin). Building on his interview, the story focused on the professionalization of prison officers. This included discussions of the increasing levels of training and education that officers received and the expansion of their role to include prisoner welfare. Finally, the sixth story was that of Anne Costello, who has taught in Mountjoy Prison since the 1980s. Her story demonstrated the expansion of the prison’s rehabilitative function, particularly through the growing provision of a wide range of educational offerings, from basic literacy to professional qualifications and university degrees. Moreover, Costello particularly emphasized the importance of education in prison as a good in its own right for prisoners and not just as a tool of rehabilitation (Anne Costello, unpublished interview by Oisín Wall, November 20, 2018, Mountjoy Prison, Dublin).

The closed and sometimes secretive nature of prisons imposed some material limitations on the exhibition, which we were able to resolve somewhat. Speirs had photographed Crawley and Gaj in the 1980s and he agreed to photograph Reynolds and Costello. Luckily there are excellent representative objects in the Mountjoy and RCPI collections. Oral histories, however, presented a difficulty. Wall interviewed prison staff, and Weston kindly gave permission to use her 2016 interview with Mara DeLacy, who had worked with prisoners with HIV. However, it was not possible to locate recordings of the former prisoners. Moreover, three were dead, and at the time, the fourth was untraceable. As a result, the exhibition risked amplifying the comparatively privileged voices of staff over prisoners. This was mitigated by printing prisoners’ verbatim quotations on the displays and by curating the gallery space to emphasize the prisoners’ stories. This mitigation strategy was successful, and less than 13.2 percent of visitor feedback forms identified solely staff members’ stories as being particularly “interesting or engaging” (Anonymous, comments from exhibition feedback card, Dublin, 2019).

The core message of *Living Inside*—that prisoners are human and therefore have a human right to health—might seem like a basic idea and might even reveal a cynical view of visitors. However, 79 percent of the respondents who completed our audience feedback forms reported that the exhibition had changed the way they thought about the treatment of prisoners. The exhibition’s core message was not mentioned in the feedback forms, and the word *human* was used only once in the exhibition in a quotation about human rights; nonetheless, the audiences’ responses conveyed the impact of the message. Responses by the audience included: “I found it engaging because prisoners are human”; “They were treated very poorly, although they committed crimes they are still human”; and “Prisoners are people too [and] are entitled to Human rights” (Anonymous, comments from exhibition feedback cards, 2019, Dublin).

## Conclusion

From gaol fevers—and now coronavirus—to mental health issues, prisoners’ health has been a perennial concern for governments, prison authorities, and prisoners and their families in the nineteenth and twentieth centuries. Although epidemics, diseases, prison conditions, and governments’ priorities have changed over time, *The Trial* and *Living Inside* highlighted the lines of continuity between historical and contemporary experiences of prison medicine for viewing audiences. The projects deployed different methodologies to engage the public in discussions about this often ignored or hidden history and had distinct aims: *The Trial* was collaborative and co-created with experts by experience, and this engagement with marginalized groups was as important as the final output and responses from the audiences. *Living Inside* was curated with a traditional top-down approach that focused on the output and audience engagement. The projects shared a common subject and intention; both brought prisoners’ mental and physical health and healthcare to a broad audience and sought to use historical research to give voice to people and stories that might otherwise have been elided or silenced. In the case of *The Trial*, using verbatim extracts was particularly effective in bringing the marginalized voices of prisoners into the public sphere (Weston 2019). While *Living Inside* used verbatim extracts and oral histories as well, it also drew on other media, such as photographs and poetry, to avoid inadvertently highlighting comparatively privileged staff voices over those of prisoners. In both projects the intersecting stigmas around imprisonment, disease, mental illness, institutional abuse, and addiction were confronted to engage audiences who might otherwise have never thought about life or health in prisons. These first-time engagements are clearly articulated in the audience comment cards: as one viewer reported, *The Trial* prompted them to think differently about the “vulnerability and lack of agency experienced by inmates. Lack of basic facilities making latent or already existent mental illness more acute. Issues around human dignity of inmates” (Anonymous, comments from artwork feedback card, Dublin, 2018). The projects encouraged visitors to engage in “ethical remembering” of a marginalized group whose vulnerabilities are often overshadowed by the crimes they have committed. They also allow historians, as John Saltmarsh and Matthew Hartley (quoted in King and Rivett 2015, 226) have argued, to link “the pursuit of knowledge with the pursuit of a healthier society”without compromising the core values of the discipline.
